# Being a better version of yourself: genetically engineered probiotic bacteria as host defense enhancers in the control of intestinal pathogens

**DOI:** 10.1080/19490976.2025.2519696

**Published:** 2025-06-18

**Authors:** Ewa Carolak, Joanna Czajkowska, Adrianna Stypułkowska, Wiktoria Waszczuk, Agata Dutkiewicz, Krzysztof Grzymajlo

**Affiliations:** Faculty of Veterinary Medicine, Department of Biochemistry and Molecular Biology, Wrocław University of Environmental and Life Sciences, Wrocław, Poland

**Keywords:** Probiotics, genetic modification, CRISPR-Cas, gastrointestinal pathogens, microbiome, gut health, biosafety

## Abstract

Intestinal pathogens pose a significant global health burden, and traditional antibiotic treatments often disrupt the beneficial gut microbiota that plays a crucial role in maintaining host health through pathogen prevention and immune regulation. Although probiotics have emerged as promising therapeutic agents, their efficacy is limited by strain-dependent variations, survival challenges in the gastrointestinal tract, and inconsistent immune responses. Recent advances in genetic engineering, particularly CRISPR-Cas systems and their combinations with complementary technologies, such as Cre-lox and RecE/T, have enabled the precise modification of probiotic strains to enhance their therapeutic potential. These enhanced probiotics demonstrate improved functionality through multiple mechanisms, including increased adhesion via the expression of specific proteins (InlA, FnBPA, and LAP), targeted antimicrobial activity through engineered sensing systems (*Lactococcus lactis* detecting *Vibrio cholerae* CAI-1), and enhanced immunomodulation through cytokine production. Results have demonstrated the potential of genetically modified probiotics in preventing and treating gastrointestinal infections through mechanisms that include competitive exclusion, bacteriocin production, intestinal barrier reinforcement, and immune modulation. However, challenges remain in ensuring genetic stability and preventing horizontal gene transfer. Future research should focus on optimizing probiotic strains for targeted applications while addressing biosafety concerns. By understanding the complex interplay between probiotics, pathogens, and host immunity, innovative strategies can be developed to harness the full therapeutic potential of probiotic interventions in maintaining gut health.

## Introduction

Intestinal pathogens such as *Salmonella enterica*, *Escherichia coli*, and *Clostridium difficile* pose a significant global health risk, leading to approximately 600 million foodborne illnesses each year and resulting in 420,000 fatalities worldwide, notably impacting children, the elderly, and individuals with compromised immune systems.^1,2^ These infections occur in the host’s gastrointestinal tract (GIT) -

a complex ecosystem where maintaining a delicate balance among the host, commensal microbiota, and potential pathogens is crucial for health.^3^ The gut microbiome – primarily made up of Firmicutes, Bacteroidetes, Actinobacteria, and Proteobacteria – plays an essential role in host homeostasis by aiding digestion, metabolism, vitamin production, and immune regulation while preventing infections through competition for resources, production of antimicrobial substances, and fortifying barriers.^4–9^

During infections, interactions among the host, pathogens, and microbiota often affect their severity.^10^ Some host defense mechanisms can be strengthened by supplementing them with nonpathogenic microbes known as probiotics.^11^ The role of probiotics in modifying the microbiota has been thoroughly studied, demonstrating promise in preventing and treating various gastrointestinal disorders.^3,12–14^ Strains of *Lactobacillus*, *Bifidobacterium*, and other beneficial bacteria aid in protecting the host during infections by bolstering gut barrier function, modulating immune responses, and competing with pathogens as essential resources.^15^ This is achieved through direct competition for adhesion sites in the intestinal epithelium, access to available nutrients, occupation of ecological niches, and alteration of the local environment through metabolite production that changes pH and oxygen levels. Nonetheless, conventional probiotics face significant challenges, including poor survival in the GIT, inconsistent colonization, and variable efficacy among different populations and conditions.^16–18^

This review concentrates on the emerging field of genetically modified (GM) probiotics, which mark a significant improvement over traditional probiotics. Through genetic engineering methods, probiotics can be enhanced to overcome their inherent limitations, providing improved stress resistance, survivability, and targeted therapeutic functions.^19–21^ The review specifically looks at genetic modifications of probiotics that strengthen the critical three-way interactions between pathogens, hosts, and microbiota. It emphasizes modifications that support the host in combating and eliminating pathogens by fortifying natural defense mechanisms, promoting beneficial microbial communities, and disrupting pathogen colonization and virulence. These modified organisms have the potential to revolutionize treatment strategies for intestinal infections while reducing reliance on antibiotics.

We analyze current strategies for probiotic genetic modification, assessing their mechanisms of action against intestinal pathogens, safety considerations, and clinical prospects. By comprehending the complex interactions within the gut ecosystem, GM probiotics may offer tailored solutions to address both the immediate threat of intestinal infections and the long-term challenge of antimicrobial resistance, which remains one of the most pressing public health issues of our time.

## Probiotic strains and gut health

Probiotics are live microorganisms that confer health benefits to the host when present in adequate amounts.^22^ These microorganisms include bacteria and yeasts of the following genera: *Lactobacillus*, *Bifidobacterium*, *Streptococcus*, *Enterococcus*, *Escherichia*, *Bacillus*, and *Saccharomyces* .^23–25^ They naturally occur in fermented foods, for example, yogurt, kefir, kimchi, and kombucha. Detailed examples of selected probiotic strains are summarized in Table 1.

**Table 1.** Most commonly used probiotic strains, their source, main effect, potential effect, and probiotic purpose.

Probiotic strain	Source	Main effect	Action	Probiotic purpose	Reference
*Lactococcus lactis*	Buttermilk, cheese production	Affects *Salmonella, Pseudomonas, Vibrio*, and *Leptospira* strains’ motility	Inhibits *Salmonella* motility by disrupting flagellar rotation	Supports gut health by reducing the harmful motility of pathogens.	Wu et al.^26^; Kudo, Morimoto & Nakamura^27^
*Lactobacillus acidophilus*	Fermented dairy products	Found in gastrointestinal tract, oral cavity	Antagonistic to *S*. *aureus, E. coli, S*. Typhimurium	Supports digestive health; combats specific foodborne and intestinal pathogens.	Gilliland & Speck^28^; Huang et al.^29^
*Lactobacillus casei*	Fermented dairy products	Lactic acid production	Inhibits *Helicobacter pylori* growth, supports gastrointestinal health	Prevents harmful bacterial overgrowth, supports digestion.	Hill et al.^30^; Cats et al. ^31^
*Lactobacillus plantarum*	Fermented vegetables	Antioxidant activity	Supports intestinal permeability, antioxidant activity	Contributes to gut barrier protection and antioxidant defense.	Kleerebezem et al.^32^; Bested, Logan & Selhub^33^
*Escherichia coli* Nissle 1917	Human gut microbiota	Competition with pathogens for iron	Supports nutritional immunity, reduces inflammation	Fights pathogenic bacteria, strengthens immune response.	Zhao et al.^34^; Deriu et al.^35^
*Bifidobacterium bifidum*	Human gut microbiota	Adhesion to the intestinal epithelium	Reduces apoptosis, improves gut health	Maintains epithelial integrity, protects against intestinal damage.	Turroni et al.^36^
*Lactobacillus rhamnosus*	Healthy female genitourinary tract	Found in fermented dairy products like yogurt	Supports gut health, reduces diarrhea, and boosts	Enhances gut integrity, reduces the risk of diarrhea caused by antibiotics and infections.	Licitra, Carpino and Donnelly^37^; de Vrese et al.^38^
*Saccharomyces boulardii*	Tropical yeast	Used in brewing, baking	Protects against pathogens, exhibits antitoxic properties	Treats diarrhea and enhances pathogen resistance in the gut.	Capece et al.^39^; Rajkowska & Kunicka-Styczyńska^40^
*Bifidobacterium longum*	Food products	Produces lactic acid	Prevents the growth of pathogens, promotes gut health	Supports a balanced microbiome and suppresses harmful bacteria.	Schell et al.^41^
*Bifidobacterium breve*	Human gut microbiota	Symbiotic intestinal bacteria	Treats diarrhea, IBS, and colds	Addresses gut issues, boosts resilience against infections.	Lyra et al.^42^
*Streptococcus thermophilus*	Yogurt, milk products	Reduces lactose in dairy products	Facilitates lactose digestion, helps lactose-intolerant individuals	Improves dairy tolerance, contributes to gut-friendly dairy production.	Ibrahim et al.^43^; Iyer et al.^44^
*Lactobacillus reuteri*	Human gut, breast milk	Reduces gut inflammation, balances microbiota	Produces antimicrobial molecules, such as ethanol, organic acids, and reuterin, and strengthens the gut barrier	Helps treat diarrhea, colic, and improves digestive health	Mu et al.^45^
*Lactobacillus gasseri*	Human gut, fermented foods	Regulates metabolism, inhibits pathogens, and supports the gut barrier	Produces lactic acid and gassericin A, enhances gut barrier integrity	Helps maintain vaginal health, relieves Helicobacter pylori infections help treat diarrhea	Selle & Klaenhammer^46^
*Bifidobacterium infantis*	Breast milk, infant gut microbiota	Reduces intestinal inflammation, enhances immunity	Digests human milk oligosaccharides, modulates the immune system	Treats IBS symptoms, supports healthy gut development, and prevents necrotizing enterocolitis	Hidalgo-Cantabrana et al.^47^
*Bacillus subtilis*	Soil, fermented foods	Enhances digestion, stimulates of the immune system	Reduces infection by Salmonella enterica serotype Enteritidis, Clostridium perfringens, and Escherichia coli O78:K80, produces digestive enzymes and antimicrobial compounds	Supports protein digestion, strengthens gut defenses	Cutting^48^; Mohamadzadeh & Abbaspour^49^
*Heyndrickxia coagulans (Bacillus coagulans)*	Soil, fermented plant products	Stabilizes gut microbiota, reduces gut inflammation	Produces lactic acid, inhibits harmful bacteria, produces spores	Eases IBS symptoms, improves digestive function	Lian et al.^50^; Cao et al.^51^
*Akkermansia muciniphila*	Natural mucus layer of the human gut	Strengthens gut barrier, regulates metabolism	Degrades mucin, produces acetate and propionate, modulates immune responses	Reduces obesity markers, improves gut integrity	Cani & de Vos^52^

For safe and effective human consumption, probiotic bacteria must meet specific criteria.^53,54^ Strains should be well-characterized, safe for intended use, and supported by positive human clinical trials.^54^ Ideally, probiotic strains should originate from the human intestinal tract and demonstrate biological activity against target organisms.^55^ Quality control measures should ensure the retention of functional health characteristics and strain stability.^56^ Additionally, probiotics must be technologically suitable for incorporation into food products and should exhibit antimicrobial activity against foodborne pathogens.^57^ These criteria help ensure the efficacy and safety of probiotic strains for human consumption.

Currently, various probiotic strains and conglomerates are available in pharmacies as capsule supplements.^58,59^ Common single-strain supplements include *Lactobacillus acidophilus* NCFM, *Lactobacillus rhamnosus* GG, *Bifidobacterium lactis* Bb-12, *Saccharomyces boulardii* CNCM I-745, and *Bacillus coagulans* GBI-30, 6086. Popular multi-strain formulations combine species like *L. acidophilus*, *L. rhamnosus*, *B. longum*, *B. bifidum*, and *L. plantarum*. Multi-strain conglomerates provide synergistic benefits by offsetting the limitations of individual strains.^60,61^

The gut microbiota, comprising approximately 10^14^ bacterial cells across more than 1000 species, functions as a critical physiological component beyond digestion.^62^ A healthy gut microbiome plays a key role in maintaining systemic homeostasis by supporting proper digestion and nutrient absorption, modulating immune responses, protecting against pathogens, and contributing to the production of anti-inflammatory and neuroactive metabolites. Probiotics play a crucial role in maintaining gut health.^63^ They modulate the gut microbiota, enhance intestinal barrier function (upregulation of tight junction proteins, such as claudin-1, occludin, ZO-1),^64^ stimulate mucin production, enhance epithelial cell proliferation,^65^ enhance competitive exclusion of pathogens, production of antimicrobial substances (bacteriocins, organic acids, hydrogen peroxide, and biosurfactants), and immunomodulation.^66–68^ Clinical studies have demonstrated the efficacy of specific probiotic strains in treating gastrointestinal disorders such as diarrhea, irritable bowel syndrome, and inflammatory bowel diseases.^61^ Additionally, probiotics have shown potential in alleviating food allergies and influencing energy metabolism.^69^ The genetic modification of probiotics can further bolster their protective effect toward gut health, which we demonstrated in this work. All of this is crucial in preventing gut dysbiosis, which is closely linked with chronic intestinal inflammation.^70^ Chronic inflammation acts as a critical driver of gut microbiota dysbiosis, characterized by reduced diversity, altered bacterial abundance profiles, and expansion of opportunistic species that perpetuate inflammatory cycles. This inflammation-driven dysregulation compromises the microbiome’s protective functions, transforming it from a defense mechanism into a pathological contributor that heightens intestinal disease through increased barrier permeability, abnormal immune signaling, and dysregulated bacterial metabolite production.^71,72^ Persistent dysbiosis disrupts bodily homeostasis, triggering both local and systemic inflammatory cascades. There is a link between gut microbiota imbalance and pathogenesis of multiple inflammatory conditions, including asthma, inflammatory bowel disease (IBD), nonalcoholic fatty liver disease, rheumatoid arthritis, and type II diabetes mellitus (excellently reviewed by Bander et al.^73^ and Zhao et al.^74^).

## Probiotic mechanisms of action against pathogens

**Figure 1.**
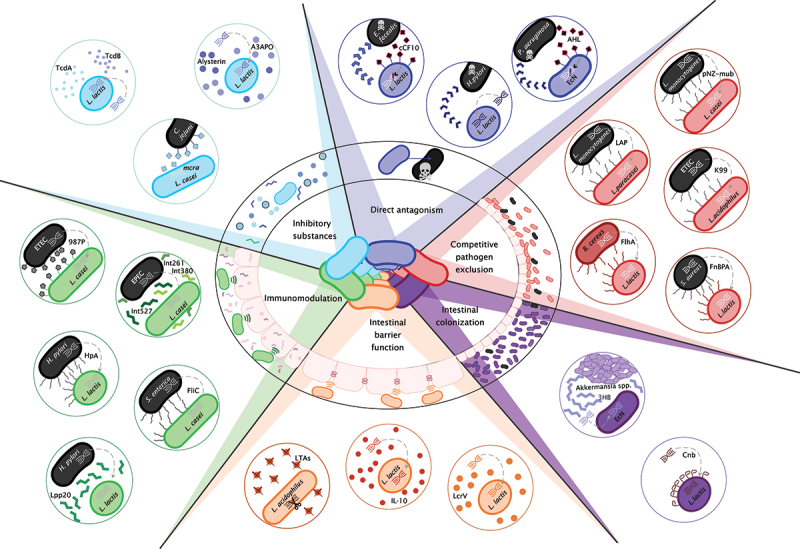
Mechanisms of probiotic bacteria against pathogens. each color represents a different strategy of the gut microbial community to fight against pathogenic species of bacteria. Examples of possible genetic modifications that may contribute to more efficient utilization of the described mechanisms are shown in bubbles in corresponding colors. All of the presented mutations are described in more detail in further chapters of the review.

Infection resistance during host–pathogen interactions usually depends on host factors and the hundreds of commensal species inhabiting the intestinal mucosa. Probiotic strains support the host’s defense against invading pathogens by limiting infections, inflammation, and pathogen-mediated damage. This protective activity can be implemented in various forms, including the production of anti-pathogen inhibitory substances, competitive exclusion, improvement of intestinal barrier function, and stimulation/regulation of the host immune response as illustrated in Figure 1.

### Direct antagonism

Some probiotic bacterial strains can directly attack pathogens by producing inhibitory substances, such as bacteriocins, which are commonly used as secondary metabolites. Bacteriocins are antibacterial or bacteriostatic molecules that often show activity only toward a given species or genus. To date, numerous bacteriocins produced by various types of microorganisms, predominantly by gram-positive lactic acid bacteria (LAB), have been described.^75^

The classification of bacteriocins is not fully established due to their diversity, although various attempts have been made to categorize them. One approach classifies them according to the producer strain – gram-negative or gram-positive bacteriocins, or more in-depth, such as for colicins/microcins, enterocins, and staphylococins^76^ produced by *Enterobacteriaceae*,^77^ enterococci,^78^ and staphylococci, respectively. Their mechanisms of action are diverse: many bacteriocins disturb cell wall synthesis by binding to lipid II or inhibiting peptidoglycan synthesis, others disrupt cell membrane integrity via depolarization or electrostatic interactions, leading to pore formation and leakage of the cytoplasm or making it permeable to specific cations, such as Na^+^ and K^+^. Some bacteriocins can be incorporated into the cell and, in turn, affect crucial enzymatic pathways within the targeted bacteria, such as replication, transcription, and protein synthesis.

Bacteriocins are considered alternatives to conventional antibiotics because of their fast mode of action, high stability under a broad range of conditions (temperature and pH), and low toxicity. Moreover, bacteriocins generally show little to no cytotoxicity against host cells. Bacteriocins are highly effective against foodborne pathogens, such as *Listeria* spp., *Salmonella* spp., *Staphylococcus* spp., *Clostridium* spp., and *E. coli*.^79,80^ For gram-negative strains, the addition of another active agent (e.g., EDTA) or the assurance of specific environmental conditions, such as a low pH, is often indispensable to enable the full activity of bacteriocins. They have been proposed for use as active substances in the food preservation industry and pharmacology. Nisin A, also known as E234, is produced by *Lactococcus lactis* and functions as a cheese preservative, as approved by the WHO and Food and Agriculture Organization. Pediocin produced by *Pediococcus acidilactici* is used as a preservative in meat products against *Listeria monocytogenes* .^81,82^ However, the *in vivo* application of bacteriocins requires further research to address important pharmacokinetic and pharmacodynamic issues. The production, activity, and novel tactics that enable the large-scale production of bacteriocins through cloning into more robust expression systems to enhance them are still under investigation. Examples of bacteriocins active against enteropathogens, along with their producer strain and mechanism of action, are presented in Table 2.

**Table 2.** The table presents examples of bacteriocins, including their class, producing strain, mechanism of action, and spectrum of activity.

Bacteriocin	Classification	Producer strain	Mechanism of action	Active against	References
Nisin	Class I – lantibiotic <5kDa	*Lactococcus lactis*	Pore-forming; inhibition of the cell wall synthesis	Gram ± (*Listeria spp*., *Staphylococcus spp*., *Bacillus spp*., *Clostridium spp. Salmonella spp*.)	Delves-Broughton et al.^83^
Lacticin Q	Class II – non lantibiotic <10 kDa	*Lactococcus lactis*	membrane permeability	Gram + (*L. monocytogenes*)	Fujita et al.^84^
Enterocin A	Class II	*Enterococcus faecium*	Form a pore in the inner membrane	Gram ± *L. monocytogenes*, *Salmonella* sp. (in combination with EDTA or low pH)	Rehaiem et al.^85^
Microcin J25	Class II – lasso peptide	*E. coli*	Inhibiting RNA polymerase and increasing superoxide production	Gram - *E.coli*, *Salmonella*	Salomon & Farias^86^
Microcin E492	Class II siderophore peptide	*Klebsiella pneumoniae*	permeabilization of the inner membrane	Gram - *K. Enterobacter, E.coli, Salmonella* sp.	Thomas et al.^87^
Colicin E1	Class I	*E. coli*	Pore formation in the inner membrane.	Gram-negative *Salmonella enterica.*	Benedetti et al.^88^
Colicin E3	Class I	*E.coli*	cleaves 16S rRNA in the 30S ribosomal subunit, inhibiting protein synthesis	Gram negative: *E. Coli, Salmonella* sp.	Ohno & Imahori^89^
Microcin M	Class IIb	*E. coli* Nissle 1917	Competition – siderophores	Gram - *Enterobacteriaceae. E. coli Salmonella sp*.	Patzer et al.^90^
Bacteriocin E 50–52	Class II	*Enterococcus. faecium*	Pore formation in the membrane	Gram - *S. enteritidis*	Svetoch et al.^91^
Pediocin PA-1	Class II	*Pediococcus acidilactici*	Pore formation in the membrane	Gram ± L*isteria* spp. *Clostridium* spp.	Papagianni & Anastasiadou^92^

Nevertheless, substances other than bacteriocins could also be produced by probiotic strains that can aid in the elimination of pathogens. For example, organic acids, such as lactic acid, help create an environment in which only strains resistant to a low pH can thrive. Short-chain fatty acids (SCFAs), such as acetic, butyric, and propionic acids, which are commonly produced by gut microbiota, especially *Bifidobacterium* spp., *Lactobacillus* spp., and *Bacteroides* spp., have a positive impact on the host metabolism and immune system. These SCFAs can also hinder pathogen colonization. For instance, they are involved in the recruitment and increase in neutrophil activity and influence the levels of released interleukins, such as pro-inflammatory IL-1β, IL-6, IL-8, and anti-inflammatory IL-10.^93^ Additionally, certain LAB strains produce hydrogen peroxide, a reactive oxygen species (ROS), by themselves or release SCFAs that can stimulate neutrophils to produce more hydrogen peroxide, resulting in a bacteriostatic effect.^94^ Another type of LAB-produced weapon is reuterin, which is produced by *Lactobacillus reuteri* and forms an intermediate during the oxidation of NADH to NAD^+^, specifically during the conversion of glycerol to 1,3-propanediol. Although reuterin has been extensively studied *in vitro*, its precise mechanism of action remains unclear. According to the principal concept, the aldehyde group of reuterin interacts with thiol groups and primary amines, thereby inactivating proteins and other molecules that contain these functional groups.^95^

### Colonization resistance

Competitive exclusion and colonization resistance refer to the competition among multiple species as essential resources.^96^ One of the primary goals of probiotic bacteria is to impede the colonization and rapid growth of pathogenic bacteria. Probiotic strains compete with pathogens for crucial resources, such as nutrients and space. Competitive exclusion and colonization resistance occur when probiotic species have a greater affinity for binding to a host cell receptor than a pathogen, affecting either the host cells directly or the local environmental conditions, such as changes in pH, nutrient concentration, and antimicrobial peptides (AMP) production.^97,98^

Nutrient availability is one of the main variables that influences gut microbiome composition. Since each strain of bacteria has different capabilities for utilizing various nutrients, they may compete for them, or a specific group may exploit available resources that another group of microorganisms cannot utilize, effectively outgrowing or crowding them out. This can result in a niche being overtaken by a particular bacterial strain. Additionally, gut microbiota can support each other’s growth through cross-feeding metabolic intermediates. The high abundance of certain probiotic strains in the gut occupies various nutrient niches, leaving them unavailable to pathogens. For example, *E. coli* strains exhibit different abilities to utilize various sugars; the *E. coli* HS and Nissle 1917 (EcN) probiotic strains can outcompete pathogenic *E. coli* O157:H due to their broader sugar utilization capabilities.^99^

Given the highly variable nature of the intestinal environment, which is dynamic and influenced by many factors, the availability of oxygen and nitrates may fluctuate over time.^100^ During inflammation or after antibiotic treatment, nitrate levels rise, creating a new niche that can be overtaken by pathogenic strains, such as *S*. Typhimurium. However, the EcN probiotic strain can also compete with *S*. Typhimurium for nitrates, limiting its growth.^101^ Keeping this in mind, the composition of gut microbiota can be altered by introducing specific nutrients and creating new niches that encourage the growth of probiotic strains while restricting that of pathogens.

The initial phase of pathogen colonization involves targeting and adhering to the intestinal epithelium; probiotics occupy these sites and block their attachment and further proliferation. This is a complex process involving the production of numerous proteins that play a role in adhesion to different surfaces, such as mucus and intestinal epithelial cells.^102^ Both probiotic and pathogenic strains possess similar structures, such as fimbriae, flagella, and surface proteins, which enhance their ability to target and adhere to host cells.^101,102^ Numerous studies have confirmed that some probiotic strains exhibit remarkable adhesion abilities and can block binding sites accessible to pathogens.^103–105^ For instance, *L. reuteri* strains strongly adhere to Caco-2 cells, effectively inhibiting the adhesion of *E. coli*, *S*. Typhi, *S*. Typhimurium, and *E. faecalis*. This adhesion may be mediated by surface proteins, such as MapA (mucus-binding protein).^104,106,107^ Another study indicated that *L. reuteri* expresses a protein on its surface that inhibits the binding of *Helicobacter pylori* to glycolipid receptors *in vitro*.^108^ Other LAB strains, including *L. rhamnosus*, *L. plantarum*, *L. custorum*, and *L. coryniformis*, demonstrated anti-adhesion effects against *E. coli in vitro*.

### Intestinal barrier function

The intestinal epithelial cell layer acts as a physical and biochemical barrier that prevents the entry of pathogens, toxins, and other environmental factors. Therefore, the integrity of this barrier is crucial for host homeostasis and protection against pathogens, as improperly formed tight junctions are among the most important sites of entry for pathogens. Among the various methods used to secure and improve the intestinal barrier, probiotics appear to be one of the most promising solutions. The major mechanisms by which probiotics improve intestinal barrier integrity, stability, and regeneration capacity are induction and reorganization of tight junction proteins,^109,110^ increased mucus secretion,^111^ stimulation of epithelial cell proliferation, and cooperation with host immune cells.^112^ Numerous studies have reported that probiotic strains can protect intestinal barriers during pathogenic infections.^113,114^ For example, members of the LAB family (*Lactobacillus casei* DN-114 001 and *Lactobacillus acidophilus*) enhance membrane barrier stability during *E. coli* infection.^110,115^ Moreover, some probiotic strains play a role not only in barrier stability but also in promoting intestinal barrier regeneration.^113^ Others, such as *L. plantarum* WCFS1, induce the production of tight junction proteins, including occludin and claudin-1, resulting in increased barrier stability.^64,116^ Commensal and probiotic bacteria can increase cellular proliferation via ROS,^117^ or in the cases of *B. thetaiotaomicron* and *Faecalibacterium prausnitzii*, modulate the mucus barrier by modifying goblet cells and mucin glycosylation.^118^

### Immunomodulation

The role probiotics play in stimulating and regulating the immune system is complex, where they can modify both local and systemic responses.^66–68^ Their action can be directed toward either activating the expression of anti-inflammatory molecules or decreasing that of pro-inflammatory molecules. For example, probiotics may influence allergic reactions by modulating the expression of IgA. Despite their well-known probiotic activity in modulating immune responses in the mammalian gut, the exact mechanisms underlying this phenomenon remain unclear.

Immune system modulation is both host-specific and probiotic strain-specific and involves various signaling pathways in the host.^119–121^ This regulation results from complex interactions between bacterial membrane structures, secreted ligands (pathogen-associated molecular patterns [PAMPs]), and host receptors recognized as pattern recognition receptors (PRRs). PAMPs are structures responsible for bacterial swimming, swarming, adhesion, and invasion, and include fimbriae, flagellins, secretion system elements, ligands, and effectors secreted directly into the host system.^122,123^ PRRs comprise Toll-like receptors (TLRs), retinoic acid-inducible gene-I-like receptors, nucleotide oligomerization domain (NOD)-like receptors (NLRs), and C-type lectin receptors.

Probiotic strains can reduce intestinal inflammation by downregulating TLRs through interference with enterocyte signaling pathways (such as NF-κB and TNF-α secretion), as well as by modulating the expression of pro-inflammatory cytokines. For example, *Lactobacillus sakei* can induce the expression of pro-inflammatory cytokines (IL-1β, TNF-α, and IL-8), whereas *Lactobacillus johnsonii* promotes the production of anti-inflammatory cytokines (such as TGF-β).^124^ Interestingly, changes in the levels of TLRs and their activation can affect the innate immune response. For example, *L. casei* activates TLR-4 in *Salmonella*-infected mice, which triggers the expression of IL-10, IFN-γ, and IL-6, and reduces the levels of TLR-α.^124,125^

In tissues and cells that do not express TLRs, NLR may assume their functions. Many probiotic strains have been shown to interfere with the immune system via NLRs, usually via the NOD signaling cascade. For example, *Lactobacillus* strains, such as *L. gasseri* and *L. delbrueckii*, increase the expression of NLRP3, leading to its activation. Moreover, *L. salivarius* has been shown to exert anti-inflammatory effects through inducing IL-10 expression via NOD2 activation.^126,127^

Another means by which probiotics interfere with the host immune system may result from the stimulation of regulatory T or B cells to increase the production of IgA, which in turn plays a crucial role in antigen recognition, toxin neutralization, and regulation of gut homeostasis.^128,129^ For example, *L. paracasei* subsp. *paracasei* NTU 101 can modulate the presentation of antigens, enhance the activation of auxiliary T cells by increasing costimulatory molecules (CD40, CD80, and CD86), increase the production of IgG antibodies in serum, and inhibit immune imbalance by increasing the expression of IFN-γ and IL-1.^130^ Moreover, *L. rhamnosus* can increase the expression of IFN-γ (Th1 cytokine) and IL-4, IL-6, and IL-10 (Th2 cytokines), which consequently increases resistance to pneumococcal infection.^131^

## Limitations of conventional probiotics

**Figure 2.**
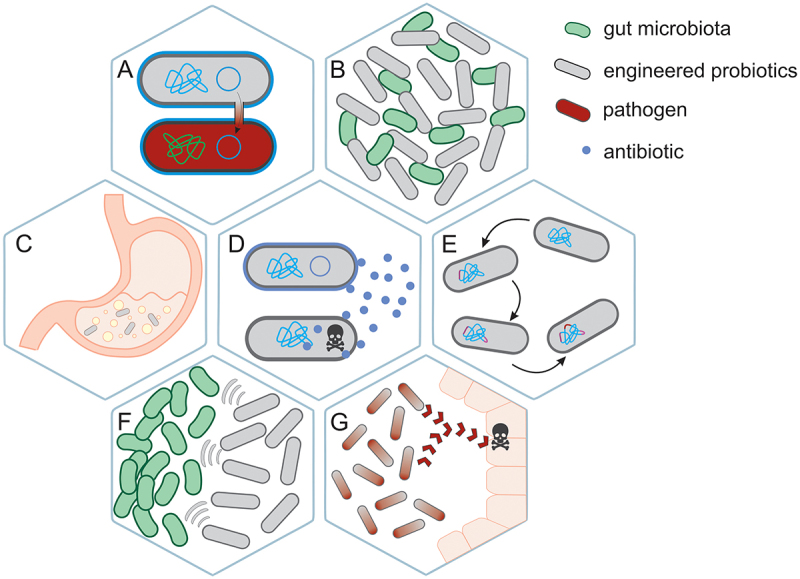
Limitations of conventional probiotics. administration of probiotics, especially engineered ones, to patients has its limitations, some of them include: (A) possibility of HGT between probiotics and pathogens, which may result in aiding pathogens, (B) overgrowth of genetically modified probiotics, and thus microbial imbalance, (C) possibility of probiotics to survive the stomach acid conditions in insufficient number to colonize the gut, (D) antibiotic resistance, that can be spread in the population of other gut bacteria, both beneficial and pathogenic, (E) DNA instability, that can result in losing the introduced mutation, or gain of unsolicited one, (F) interactions with gut microbiota, that may result in disrupting homeostasis, (G) gain of pathogenic activity by administered probiotics.

Current therapeutic applications heavily rely on naturally derived probiotic strains that are available as supplements and medications (see above). These probiotics, although beneficial, have notable limitations^11,132,133^ (Figure 2). They can partially restore gut microbiota after antibiotic therapy; however, their therapeutic potential remains restricted due to impaired gastrointestinal survivability under acidic conditions (pH < 3.0) and bile exposure, which significantly reduces viable bacterial count. Colonization remains predominantly transient with minimal microbiome integration, as evidenced by rapid post-supplementation clearance. Conventional probiotic strains often fail to effectively combat specific gut pathogens that they do not significantly inhibit or displace.^134^ In mouse models, certain probiotics, like *L. salivarius* and *B. infantis*, failed to enhance anti-inflammatory responses, highlighting inconsistent immune benefits.^135^ In acute, severe dehydrating diarrhea, probiotic therapy showed limited benefits, with no significant reduction in diarrhea duration or stool output observed in a study using *Lactobacillus* GG.^136^ Probiotics often only provide supportive benefits rather than targeted therapeutic effects, making them insufficient for addressing specific pathogenic infections or distinct microbial imbalances. Some research suggests their effects may be ambiguous and, in certain situations, even detrimental. A study on patients with predicted severe acute pancreatitis found that conventional probiotics did not reduce infectious complications and were linked to higher mortality in the probiotic group versus the placebo group, highlighting serious safety concerns in critically ill populations.^137^

Factors that affect efficacy, such as strain type, dosage, and administration route vary among individuals. Probiotics face challenges in surviving technological and gastrointestinal stress, and their broad, nonspecific actions can reduce their targeted effectiveness. Further, safety concerns and inconsistent regulatory oversight complicate their use.^21,134,138,139^ Individual response variability presents a significant issue, as host-specific factors like genetic polymorphisms and indigenous microbiota composition substantially influence probiotic effectiveness. Additional variability stems from strain selection, dosage formulation, and administration routes, with optimal therapeutic parameters remaining poorly defined despite variations in colony-forming unit (CFU) counts across products.

Recent developments have focused on enhancing probiotic capabilities using various strategies, including exploring next-generation probiotics and genetic engineering approaches. Emerging approaches include non-conventional strains and exploration of next-generation probiotics (NGPs) like *Akkermansia muciniphila*, *Faecalibacterium prausnitzii*, and *Bacteroides fragilis*, which offer potential for individualized treatments and improved safety profiles.^140^ These “smart probiotics” aim to simulate the benefits of fecal microbiota transplantation while using characterized gut colonizers. However, viable intestinal delivery remains a significant challenge due to the stringent survival conditions required by these new strains. Understanding probiotics’ mechanisms of action and improving their therapeutic potential through targeted modifications could expand their application beyond supportive care. Genetic engineering of strains, such as those of lactobacilli and bifidobacteria, offers the potential to enhance their efficacy and safety; however, this requires rigorous safety assessments and clear regulatory frameworks. Addressing these limitations using bioengineered probiotics may enable more effective and specific therapies for gut health and foodborne pathogen control.^141,142^

## Safety aspects of genetically modified probiotic strains

The safety of probiotic strains is of paramount importance, and guidelines for safety assessments are thus well documented.^143,144^ The most commonly used bacterial species (*Lactobacillus* and *Bifidobacterium*) are not harmful to human health. Although generally considered safe, probiotics may pose a risk to certain populations when inappropriately administered. Critically ill and hospitalized patients, infants, the elderly, immunocompromised individuals, and those with specific medical conditions are most vulnerable to adverse effects.^145,146^ Potential risks include systemic infections, fungemia, gastrointestinal ischemia, and endocarditis.^147,148^ Concerns include the possibility of deleterious metabolic effects, excessive immune stimulation, and genetic stability of the strains over time.^148,149^ Further concerns have been noted regarding opportunistic pathogenicity in vulnerable groups and the possibility of antibiotic resistance transfer to gut pathogens.^145^ Although the long history of probiotic use and clinical trials supports their safety and has demonstrated their benefits in various settings, caution should be exercised when administering probiotics to at-risk populations.^146,148^ Therefore, the World Gastroenterology Organization recommends restricting probiotic use to strains with proven efficacy.^150^

Additionally, integrating GM probiotics into consumer products requires further safety evaluations to ensure that they do not pose risks to human health or the environment. Key safety aspects of GM probiotic strains include (I) genetic stability, (II) potential pathogenicity, (III) antibiotic resistance, (IV) horizontal gene transfer (HGT), (V) metabolic activity, (VI) interactions with the gut microbiota, and (VII) microbial biocontainment systems.

- Genetic stability is crucial to ensure the safety and functionality of probiotics. Stable genetic modifications prevent the emergence of unwanted traits and ensure the retention of beneficial properties. This stability is essential for large-scale production, allowing the strain to yield high output in cost-effective growth media.^151^ Additionally, probiotics must remain stable as concentrated starter cultures and in final products, such as supplements or fermented dairy items. Strains are often engineered through chromosomal integration of introduced genes, rather than by relying on plasmid-based vectors, which are more prone to loss and HGT. Regular monitoring and rigorous testing under various environmental conditions are necessary to assess stability over time, ensuring that probiotics consistently deliver their intended health benefits without reverting to a pathogenic state or losing functionality.^152,153^
- In addition to their genetic stability, the potential pathogenicity of GM probiotic strains is a significant safety concern. Although the parental strains used for genetic modification are typically well-characterized and safe, genetic manipulation could inadvertently introduce or activate virulence factors.^154^ Each bioengineered strain must undergo a thorough evaluation in the presence of virulence factors, which are genes that can potentially cause disease. These genes, along with those encoding enzymes involved in the synthesis of toxic or allergenic compounds and their precursors, should be excluded. The introduction of foreign DNA can lead to the production of new substances, including proteins or metabolic products that might have toxic or allergenic effects.^21,154^ It is essential to compare the amino acid sequences of newly expressed proteins with those of known proteins to identify potential homologies. Additionally, their anti-nutrient activities, such as those of protease inhibitors or lectins, as well as their stability under heat, processing, and degradation conditions, must be assessed in appropriate gastric and intestinal model systems. Nevertheless, no definitive tests are available to predict allergic responses to these newly expressed proteins in humans.^154^
- Over 68% of probiotics isolated from various products have been reported to exhibit resistance to two or more antibiotics.^155^ LAB have naturally acquired resistance to certain antibiotics.^154^ However, this intrinsic resistance is typically nontransferable (chromosomally encoded) to other bacteria (including pathogens) present in the intestinal environment, indicating that these bacteria remain susceptible to most antibiotics used in clinical treatment. Consequently, nontransferable resistance does not generally pose a significant safety concern. Conversely, transferable antibiotic resistance (when located on mobile genetic elements such as plasmids, transposons, and integrons) is a more serious issue due to the potential of this resistance spreading to pathogenic bacteria. This is particularly concerning for enterococci, as some strains possess plasmids encoding genes for antibiotic resistance; transfer of these plasmids has been demonstrated during food fermentation and *in vivo* .^156^ In particular, concerns have been noted regarding enterococcal conjugative resistance to glycopeptide antibiotics, such as vancomycin and teicoplanin, as vancomycin is one of the last-resort antibiotics used for treating certain multidrug-resistant pathogenic microorganisms.^157^ Glycopeptide resistance is more frequently found in *Enterococcus faecium* than it is in *Enterococcus faecalis*, with the latter being more common in human feces. Among enterococci isolated from human infections, *E. faecalis* predominates, highlighting the importance of monitoring and controlling antibiotic resistance in probiotic strains to prevent the spread of resistance to pathogenic bacteria. Therefore, European Food Safety Authority guidelines consequently require demonstration that resistance is chromosomally encoded rather than plasmid-mediated, with a specific focus on the absence of transferable genes conferring resistance to clinically relevant antibiotics, such as vancomycin and tetracycline before granting Qualified Presumption of Safety status.^158^
- The issue of HGT is closely related to antibiotic resistance, which involves the movement of genetic material between organisms, rather than through vertical transmission (from parent to offspring). The use of *Lactobacillus* probiotics carries the risk of horizontally transferring antibiotic resistant genes to pathogenic bacteria. Although research in this area is still in its early stages, the presence of resistant genes on mobile genetic elements could facilitate the acquisition of these genes through bacterial conjugation.^159^ Although evidence of the spread of antibiotic resistance via gene transfer in probiotics is limited, uncertainty surrounding the *in vivo* transfer of resistant genes from *Lactobacillus* probiotics to pathogenic bacteria has led to the frequent use of *in vitro* conjugation models.^160^ *In vitro* and *in situ* studies have often reported the plasmid transfer of antibiotic resistance genes, particularly those encoding resistance to tetracycline and erythromycin, from *Lactobacillu*s probiotics to pathogenic bacteria.^160^ Despite some evidence suggesting that food probiotics and human gut microbiota can serve as reservoirs for antibiotic resistant genes, conclusive findings are still pending, and further clinical studies are required.^161^
- Traditionally, harmful metabolic activity has not been observed in established probiotic strains but must be assessed in GM probiotics. For instance, mucus degradation capacity, considered a marker for invasive potential, has not been detected in specific commercial probiotics but has been found in some GM strains.^156^ Another concern is metabolic activity related to bile salt deconjugation. Secondary bile acids resulting from this process may exhibit carcinogenic properties.^162^ Therefore, probiotics with a high capacity for bile salt deconjugation and dehydroxylation require careful evaluation despite the potential benefits they have for managing cholesterol levels.^163^ In addition, other virulence factors that require careful evaluation include the ability of the probiotic strain to resist complement-mediated killing, which could enhance its survival in the bloodstream, and the formation of a capsule that might protect it from phagocytosis.^162^
- Probiotics have been demonstrated to interact with other microorganisms, and some were found to inhibit the adhesion of pathogens to the human intestinal epithelium.^164^ However, interactions with certain probiotic strains have also been reported to increase the adhesion of specific pathogens to human and animal mucus. The biological significance of these increased adhesion events requires further evaluation, as they could pose additional risks associated with certain probiotic strains. The potential “crosstalk” between probiotics and intestinal cells must also be considered. This includes not only interactions within the healthy intestine but also specific interactions and immune responses in cases of intestinal injury or gastrointestinal disease.^163^ Although current knowledge in this area is limited, research on the interactions between microbes and intestinal cells is ongoing. New findings in this research area could help establish the mechanisms underlying probiotic action and identify potential safety risks. For example, some commensal bacteria, such as *Enterococcus faecalis*, have recently been shown to trigger epithelial cell trafficking of a protein that serves as a receptor for *S*. Typhi, potentially increasing host susceptibility to typhoid or enteric fever.^165^ As some enterococci are used as probiotics, this strain-dependent phenomenon could be considered a risk factor and thus warrants further investigation.
- Microbial biocontainment systems aim to prevent the uncontrolled spread of genetically modified microbes or their genetic material into the environment. These concerns are significant not only for the clinical use of probiotics but also for the agricultural and industrial uses of GM. To date, several techniques have been implemented to tightly control the proliferation of GM outside of the authorized conditions. These techniques include biocontainment through autotrophy, conditional essentiality, toxins, or microbial kill switches.^166,167^ Recently, CRISPR-based kill switches have been developed in E. coli Nissle 1917, allowing EcN proliferation in the gut while enabling cell death to be initiated by the consumption of an inducer and subsequent excretion from the host body. Moreover, this technique could be adapted to other probiotic strains as it exploits the CRISPR/Cas9 and TetR/Ptet systems applicable to them.^168^ However, even after cell death, ensuring the safety of genetic material remains crucial. Several approaches have been proposed, including toxin-antitoxin (TA) modules, conditional replication origins, and CRISPR-Cas-based systems. TA modules rely on the expression of a lethal toxin gene along with a corresponding antitoxin gene, where the antitoxin prevents the translation or activity of the toxin, thereby promoting plasmid retention. Conditional replication origins, such as ColE2-P9, protect genetic material by requiring the initiator protein Rep2 for plasmid DNA replication. The CRISPR-Cas system offers protection even for genes located on the chromosome, as it targets and cleaves specific DNA sequences, enabling targeted gene degradation in case of unauthorized release.^169^ Nevertheless, microbial and genetic biocontainment systems are still under development, and the lack of clear guidelines requiring their implementation remains a significant challenge. However, the increasing use of genetically modified organisms makes the development of such regulations essential.

Overall, safety assessment guidelines must consider the diversity of microorganisms and identify specific risks associated with each probiotic strain, as well as the potential interactions that can occur among probiotics, hosts, and food components. Epidemiological surveillance and follow-up studies of novel strains are crucial for maintaining high safety standards for probiotics. Monitoring the positive health and potential side effects of probiotics is necessary to ensure their continued safe use. Therefore, regulatory frameworks governing the use of GM probiotics have been designed to ensure their safety for human consumption and environmental release. Regulatory bodies, such as the US Food and Drug Administration, the European Food Safety Authority, and the WHO, have established stringent guidelines for the development, testing, and commercialization of GM probiotics. Compliance with these guidelines is essential to ensure the safe use of GM probiotics in consumer products.

## Tools for chromosomally editing probiotic strains

*Lactobacillus* and *Bifidobacterium* species have long been utilized as probiotics and are now gaining attention as living diagnostic and therapeutic agents that have the potential to assess and promote human health. Bioengineering of these species for biomedical purposes remains underdeveloped compared with that of other probiotic strains, such as EcN, especially in the case of *Bifidobacterium* species.^170^ Although robust genetic tools were developed early for gram-negative bacteria, the thick cell wall structures and genetic resistance that gram-positive bacteria have can hinder foreign DNA uptake and complicate genetic manipulation.^171,172^ However, recent advancements have significantly improved the genome editing techniques that can be used for genetically challenging microorganisms, offering new opportunities for their modification and application in therapeutic contexts.^21,141,170,173^

Initial efforts focused on basic transformation techniques, with plasmid-based systems being one of the first genetic tools to be applied to gram-positive bacteria. These plasmids were derived from those naturally occurring in LAB, and their introduction into *Lactobacillus* species has laid the groundwork for future genetic manipulation. However, the transformation efficiency remains low owing to the physical barriers posed by the cell wall, and most research has been limited to a few strains.^174^ Homologous recombination techniques have been adapted for use in gram-positive probiotics, allowing for more precise genome modifications. As shown in Table 3, several variations of this approach have been developed, including non-replicative plasmid-mediated homologous recombination for *Bifidobacterium*, temperature-sensitive plasmid systems, the pORI system for gene knockouts, and pTRK suicide plasmid systems. The successful application of these techniques is evidenced by achievements, such as the knockout of the tetW gene in *B. animalis* NCC2818, resulting in approximately a 50× increase in sensitivity to tetracycline compared with that of the wild-type strain.^170^ However, the lack of efficient selection markers and limited understanding of gene regulation in *Lactobacillus* and *Bifidobacterium* have hindered their progress. The use of site-directed mutagenesis and gene knockouts often yields inconsistent results due to strain-specific differences in transformation protocols and genome stability.^210^

**Table 3.** An overview of selected genetic modification tools and techniques used in probiotic bacteria, including their process mechanisms, advantages, disadvantages, and practical applications across various bacterial strains.

Editing tool	Tool description	Process overview	Advantages	Disadvantages	For	Probiotics example	Application example	References
Non-replicative plasmid-mediated homologous recombination	Non-replicative plasmid-mediated homologous recombination is a directed mutagenesis technique specifically developed for modifying genes in *Bifidobacterium*. The method utilizes a plasmid that cannot replicate within the host organism but carries both a modified version of the target gene and a selectable marker gene.	Construction of a non-replicating plasmid containing the modified target gene fragment and marker gene is followed by transformation into bacteria cells through electroporation. Homologous recombination facilitates DNA exchange between the plasmid and bacterial chromosome, resulting in integration. Successful transformants can be selected using the marker gene, ensuring permanent incorporation of the desired mutation into the bacterial genome.	Single-step transformation process	Dependent on transformation efficiency; low success rate; mutations are often unstable; residual antibiotic markers remain after modification	Gene insertion; gene deletion	*Bifidobacterium breve;Lactobacillus sake;Lactobacillus plantarum;Lactobacillus casei* *Bifidobacterium animalis*	*tetW* gene knocked out from *B. animalis* NCC2818 – approximately 50× increase in sensitivity to tetracycline compared with that of the wild-type strain	Fukiya et al.^170^; F.^175^
Temperature- sensitive plasmid-mediated homologous recombination	Method using temperature-sensitive (Ts) plasmids to facilitate precise chromosomal modifications through controlled homologous recombination. The tool enables selective gene knockout by leveraging plasmid instability at high temperatures and strategic sequence integration.	The process involves introduction of the methylated Ts plasmid to bacteria, and when shifted to nonpermissive temperatures, the plasmid cannot replicate, forcing integration into the host chromosome through homologous recombination to maintain expression of the selection marker. This system facilitates both single-crossover and double-crossover events, making it an efficient method for generating gene knockouts even with low transformation efficiencies. The successful recombinants are then selected on appropriate media at high temperatures.	Seamless editing and stable mutation	Independent of transformation efficiency; time-consuming and requires significant effort; constrained by the narrow host range of the donor plasmid	Gene knockout; single-crossover homologous recombination	*Lactobacillus gasseri*; *Bifidobacterium adolescentis*; *Lactobacillus acidophilus*; *Bifidobacterium longum*	Gene knockout mutants of the *pyrE* region of *B. longum* 105-A	Russell & Klaenhammer,^176^ Sakaguchi et al.^177^
Homologous recombination using the pORI system	Plasmid-based system involving a non-replicating plasmid (pORI) carrying a gene of interest flanked by homologous sequences.	This method leverages a plasmid that lacks an origin of replication in the target host, ensuring that the plasmid cannot replicate independently. Instead, it relies on homologous recombination to integrate its genetic material into the host genome.	Effective regardless of the transformation efficiency of the host cell; supports the growth of engineered strains at their optimal temperatures; efficient recovery of stable integrants; useful for deleting any nonessential gene across a diverse range of bacterial species	Single-crossover homologous recombination mutations require antibiotic selection for stability; reusing the same selection marker for multiple mutations is not possible; targeting genes in an operon may cause downstream polar effects	Gene knockout; gene insertions; promoter replacements	*Lactococcus lactis*; *Lactobacillus acidophilus*; *Lactobacillus gasseri*	Expression of the *pip* gene (proline iminopeptidase) in *Lactococcus lactis*	Law et al.^178^; Leenhouts, Kok & Venema^179^
Suicide plasmids – pTRK system	The pTRK system is a temperature-sensitive suicide plasmid-based genome editing tool. It enables targeted gene modifications through homologous recombination. Key features include: *upp* counter-selectable marker for marker-less editing, selectable markers, and optional transposon sequences for genome integration.	Plasmid construction includes homologous regions, selectable markers, and *upp*, which are introduced via electroporation or conjugation. Temperature shift for nonpermissive conditions prevent plasmid replication. Recombination: single-crossover event integrates the plasmid; double-crossover events removes it, leaving the edits. Selection via antibiotics, and counter-selection recovers mutants, as confirmed through PCR or sequencing.	Transformation efficiency is not a limiting factor; operates within a temperature range suitable for thermophilic lactobacilli; allows marker-free gene replacement	Low efficacy; high false-positive rates; introducing multiple mutations into a strain is not feasible when using the same selection marker	Gene deletions; gene insertions; gene knockouts	*Lactobacillus acidophilus*; *Lactobacillus gasseri*; *Lactobacillus casei*; *Lactococcus lactis*; *Lactobacillus plantarum*	Integration and expression of a porcine rotavirus capsid protein in *Lactobacillus casei* ATCC 393	Goh et al.^180^; Spangler et al.^181^; Yin et al.^182^
Two-plasmid integration system	The system enables targeted chromosome modifications through controlled homologous recombination and employs two complementary plasmids: a donor plasmid and plasmid with essential recombination machinery (adaptation of the pVE6007/pORI19 system).	The first plasmid (integration vector) contains the gene of interest flanked by sequences homologous to the target site on the bacterial chromosome. The second plasmid (recombination helper) carries the necessary recombination machinery, such as recombinases and selection marker.	Independent of transformation efficiency; supports growth at optimal temperatures; enables effective recovery of stable integrants; applicable to various species	Plasmid DNA and antibiotic markers remain integrated into the chromosome	Gene knockout; integrates expression cassettes into preferred sites in the chromosome	*Lactobacillus acidophilus*; *Lactobacillus gasseri*	Inactivation of the *L. gasseri gusA* gene (β-glucuronidase)	Russell & Klaenhammer^176^; Van Pijkeren et al.^183^
Plasmid-based allelic exchange	Introduces precise modifications into the genome by replacing a native allele with a mutated or desired allele. This method relies on homologous recombination between the host genome and a plasmid vector containing the desired genetic change flanked by regions homologous to the target locus.	A plasmid engineered with the desired genetic sequence flanked by homologous regions to the target locus in the genome is introduced into bacterial cells. Homologous recombination between the plasmid and bacterial chromosome facilitates integration of the desired allele into the genome. Counter-selectable markers or systems, such as the *sacB* gene, are used to select against cells retaining the plasmid.	Genetic changes can be introduced without leaving markers in the genome	The efficiency of homologous recombination in *Lactobacillus* can be low, and some species are naturally resistant to transformation	Gene knockouts; point mutations; gene insertions	*Lactococcus lactis*; *Streptococcus* *salivarius* ssp. *thermophilus*; *Enterococcus faecalis*; *Lactobacillus plantarum*	Lactate dehydrogenase (*ldhD*) gene deletion in *Lactobacillus plantarum*	Leenhouts et al.^179^; Maguin et al.^184^; Okano et al.^185^
Selection/counter-selection marker system	The selection/counter-selection marker system is a genetic tool that uses genes such as *upp* or *mazF* to facilitate chromosomal modifications in lactic acid bacteria.	During the process, cells are transformed with a marker system, whereafter successful transformants are selected. Next, desired genetic changes are introduced alongside the marker, followed by a counter-selection step where specific conditions are applied to eliminate cells still containing the marker, which ensures the survival of only successfully modified cells. This is followed by verification of the surviving cells to confirm the intended genetic modifications.	Efficient gene deletions and integrations at specific locations, markers (e.g., *upp*) can be reused multiple times; final modified strains are free from selection markers and plasmids; stable double-crossover mutants can be obtained	Optimization of MazF toxin expression; *upp* is present in most organisms that display nucleotide metabolism; the counter-selection agent, 5-fluorouracil, can be toxic even to *upp* mutants; requires the identification of effective counter-selection markers for optimization	Gene deletion; gene insertion; gene knockout	*Lactobacillus plantarum* 423; *Enterococcus mundtii* ST4SA; *Lactobacillus casei*; *Lactococcus lactis*; *Lactobacillus acidophilus*	Insertion of sizable DNA segments and removal of the bacteriocin genes from *Lactobacillus plantarum* 423 and *Enterococcus mundtii* ST4SA	Van Zyl et al.^186^; Song^187^
Inducible plasmid self-destruction	The inducible plasmid self-destruction (IPSD) is a tool for the genome engineering of bacteria, such as lactobacilli and bifidobacteria. This method is based on the introduction of a plasmid that self-destructs under the influence of an inducer, which facilitates the selection of homologous recombination events.	Plasmid containing an antibiotic resistance gene, replicon, and recombinase gene is introduced into bacterial cells. Through single-crossover integration using a homologous chromosomal fragment, the plasmid becomes part of the bacterial chromosome. Recombinase induction then triggers plasmid self-destruction via replicon excision. Antibiotic selection identifies cells with successful homologous integration, and subsequent growth without antibiotics isolates double crossing-over mutants that have eliminated the antibiotic resistance gene.	Independent of transformation efficiency; reliable mutations; seamless editing; self-eliminating plasmid system that does not leave behind antibiotic resistance genes; increased efficiency in the genome engineering of probiotic bacteria	Extensive mutant screening; requires careful optimization of induction conditions	Gene knockout; gene insertion	*Lactobacillus gasseri*; *Lactobacillus paracasei*; *Lactobacillus plantarum*; *Lactobacillus acidophilus*; *Lactobacillus sakei*; *Bifidobacterium longum*; *Bifidobacterium lactis*	Removal of the *tetW* gene encoding tetracycline resistance *in B. longum* IF3 − 53 to increase the safety of the probiotic; stable chromosomal integration of the LpKatL catalase gene from L. *plantarum* into *B. longum* NCC 2705 to increase its resistance to oxidative stress	Zuo et al.^188^; Zuo et al.^189^
ssDNA recombineering	Homologous recombination of single-stranded linear DNA mediated by the λ-Red enzymes, which facilitate targeted genetic alterations in bacterial genomes and enable direct chromosomal modifications with high efficiency and minimal cellular disruption.	A single-stranded DNA (ssDNA) template with desired genetic modifications that induce the expression of λ-Red recombineering proteins (Gam, Exo, and Bet) in target bacteria is electroporated into competent cells. The λ-Red machinery facilitates precise homologous recombination, enabling direct chromosomal modification without requiring antibiotic selection markers.	Low cellular toxicity; efficient point mutations; highly efficient for precise genetic modifications; enables mutant selection without requiring antibiotic markers	Dependence on transformation efficiency; works best with small genetic changes; requires precise optimization for each bacterial species; specialized protein expression	Directed point mutations; deletions up to 10 kb; 10–20 base insertions	*Lactobacillus reuteri;* *Lactococcus lactis;* *Lactobacillus* *plantarum;* *Lactobacillus gasseri*	Construction of non-selected targeted mutations on the chromosomes of *L. reuteri* and *L. lactis*	Van Pijkeren & Britton^190^
dsDNA recombineering	Double-stranded DNA (dsDNA) recombineering – a method that uses phage-derived proteins (typically the λ Red system) to precisely modify bacterial genomes through homologous recombination. Allows the integration of linear DNA fragments into specific genomic locations without requiring restriction enzymes or ligases.	Process begins by expressing λ Red proteins in host bacteria and preparing linear dsDNA with homology arms matching the target sequence. The bacteria are then made electrocompetent, transformed with the linear DNA fragment, and allowed to recover before selecting successful recombinants using antibiotics or screening methods. The entire procedure takes approximately 3–4 days to complete.	Insertion of larger DNA fragments (up to several kb) compared with when ssDNA is used; no need for restriction enzymes or ligases, reducing cost and complexity; more precise than traditional cloning methods are due to directed homologous recombination; can introduce multiple changes simultaneously	Lower efficiency compared with that of ssDNA recombineering (about 10–100× less efficient); requires expression of all three λ Red proteins – more complex than ssDNA methods are; requires longer homology arms (>35 bp vs. 20–30 bp for ssDNA); limited host range	Gene deletions; gene insertion; gene replacement	*Lactobacillus* *plantarum*; *Lactobacillus casei*	Removal of a DNA fragment: the *gnp* gene, *ldhD*, or the *glg* operon; gene insertion: the *gusA* gene from *E. coli* in place of the *ldhD* gene; allelic exchange: introducing the lox66-cat-lox71 cassette in place of the *gnp* gene	Wang et al.^191^; Xin et al.^192^; Yang et al.^193^
ssDNA recombineering + CRISPR-Cas9	Combination of dsDNA recombineering with CRISPR-Cas9 technology, where synthetic DNA fragments work with host homologous recombination machinery to introduce specific changes while CRISPR-Cas9 creates targeted double-strand breaks (DSBs) that serve as a selection tool.	A synthetic dsDNA fragment containing the desired modifications is introduced into bacterial cells, where host recombination machinery incorporates it into the genome. Simultaneously, the CRISPR-Cas9 system targets the unedited locus, creating DSBs to eliminate cells lacking the modification. This process involves designing guide RNAs (gRNAs) to target the desired genomic site, constructing a dsDNA template with the required changes, and transforming the cells with both components. After homologous recombination integrates the changes, the edited strains are selected and verified.	Higher stability of dsDNA templates; more efficient for larger genetic modifications; reduced risk of template degradation; better performance in some bacterial species; supports more complex genetic engineering strategies marker-free	Lactobacilli exhibit varied responses to CRISPR-Cas9-induced DSBs, making it challenging to predict the efficiency of the editing process; lower insertion efficiency compared with hat of the ssDNA method; more complex template preparation; higher potential for off-target effects; increased technical complexity; greater sensitivity to template design and quality	Point mutations; deletions; gene insertions; gene replacements	*Lactobacillus plantarum*; *Lactobacillus brevis*	*Lactobacillus plantarum* – insertion of genes involved in *N*-acetylglucosamine production	Zhou et al.^194^; Huang et al.^195^
dsDNA recombineering + CRISPR-Cas9	The combination of ssDNA recombineering and CRISPR-Cas9 technology provides a highly accurate genome editing technique for bacteria. This method utilizes ssDNA oligonucleotides along with CRISPR-Cas9 to enable targeted genetic alterations.	Mutagenic ssDNA oligonucleotides are designed with the desired mutation and introduced into bacterial cells along with recombinase proteins to facilitate DNA integration. The CRISPR-Cas9 system is used to target a specific genomic region, and the oligonucleotides inserted via recombineering. CRISPR-Cas9 then helps eliminate cells with unmodified wild-type sequences. Finally, the genetic modification is verified and confirmed through PCR or sequencing.	High editing efficiency (46–100%); precise genome modifications; no requirement for phenotypic selection; minimal off-target effects through optimization; applicable across multiple bacterial species; time-efficient – can be completed within 72 h.	To ensure minimal off-target effects, careful adjustments of parameters, such as plasmid copy number and spacer sequence length, are required; stability of the plasmids used could be improved	Point mutations; gene deletions; gene insertions	*Lactobacillus reuteri*; *Lactococcus lactis*; *Lactobacillus gasseri*; *Lactobacillus plantarum*	*Lactobacillus plantarum* – insertion of genes involved in *N*-acetylglucosamine production	Guo et al.^196^; Zhou et al.^194^
CRISPR-Cas9	CRISPR-Cas9 is a powerful genome-editing tool derived from a bacterial immune system, which uses a gRNA to direct the Cas9 nuclease to a specific DNA sequence where it creates a DSB.	The gRNA pairs with the target DNA sequence, and the Cas9 enzyme cuts the DNA at this location. The cell then repairs this break, often incorporating or deleting segments of DNA in the process.	High specificity, efficiency, and versatility; capable of multiple edits at once; marker-free selection	Potential for off-target effects and lethal DSBs; ethical and regulatory concerns in some applications	Gene deletions; gene insertions	*Lactobacillus crispatus*; *Lactobacillus plantarum*; *Lactobacillus reuteri*	Editing of *Lactobacillus* to enhance its resistance to stomach acids and bile salts; introduction of genes into *Bifidobacterium* to produce vitamins or other beneficial compounds	Hidalgo-Cantabrana et al.^197^; Myrbråten et al.^198^
CRISPR/Cas12a	CRISPR/Cas12a (Cpf1): this variant of the CRISPR system offers an alternative to Cas9, with different target sequence requirements and potentially reduced off-target effects.	Process is analogical to that of the CRISPR/Cas9 method, with the significant difference involving Cas12a cutting DNA at specific regions while leaving the 5′-overhangs and promoting a more uniform repair of the genome.	Easier modification of AT-rich genomes in comparison with that of CRISPR/Cas9; less toxic to bacteria than Cas9 is	In comparison with the Cas9 system, it has a lower affinity to CG regions	Multiplex gene editing	*Bacillus subtilis*; *Lactobacillus rhamnosus*	CRISPR/Cas12a in a mercury detection system – provides signal amplification	Paul and Montoya,^199^; Zhang et al.,^200^; Wu et al.^201^
CRISPRi	The CRISPR interference (CRISPRi) system is a genome editing tool that requires dead cas9 nuclease and gRNA to be incorporated into a target gene. Unlike CRISPR-Cas9, which induces DSBs for genome editing, CRISPRi uses a catalytically inactive Cas9 (dCas9) to repress gene expression by blocking transcription.	The CRISPRi system utilizes a catalytically inactive Cas9 protein, which retains its ability to bind to specific DNA sequences guided by a sgRNA but lacks the ability to cleave DNA. The dCas9-sgRNA complex binds to the promoter or coding regions of a target gene, preventing the binding of RNA polymerase and other transcription factors, which effectively silences gene expression, allowing for precise and reversible control of gene activity without altering the DNA sequence.	Simple to downregulate any gene of interest with dCas9; enables multiplexing without requiring a selection marker; gene repression via CRISPRi is reversible and does not alter the DNA sequence, reducing potential off-target effects and genomic instability	The level of gene repression can vary significantly between different target sites; only partial repression rather than complete silencing for some genes; may not work well for genes with high expression levels and those encoding long half-life proteins	Gene regulation – suppressing the expression of target genes	*Lactobacillus* *plantarum*; *Lactococcus lactis*	Silencing the *upp* gene, which encodes uracil phosphoribosyltransferase, resulting in a more than 50-fold decresase in *upp* mRNA transcription	Qi et al.,^202^; Berlec et al.^203^
CRISPR- Cas9D10A nickase-assisted plasmid toolbox (pLCNICK)	The CRISPR-Cas9D10A system is a modified version of the CRISPR-Cas9 genome editing tool designed to improve specificity and reduce off-target effects.	The Cas9D10A variant is a nickase that introduces single-strand breaks (nicks) instead of DSBs. When paired with two gRNAs that target adjacent sequences on opposite strands, it can create gradual nicks that result in a DSB at the target site.	Fast, effective, and accurate method for genome editing; low risk of chromosomal rearrangements; marker-free; avoids lethal DSBs by utilizing alternative repair pathways for nicks	Not effective for large deletions; can be less efficient than the traditional CRISPR-Cas9 system is for certain edits; can result in significant lethality due to DSBs caused by Cas9/sgRNA	Gene deletion; gene insertion	*Lactobacillus casei*; *Lactobacillus acidophilus*; *Lactobacillus gasseri*; *Lactobacillus paracasei*	Insertion of an enhanced green fluorescent protein (eGFP) expression cassette at the LC2W_1628 locus in *L. casei*	Song et al.^204^
Prophage- associated recombinases (LCABL_13040–50–60)	This tool is based on the use of homologous recombination and Cre-lox recombination. The key element is the recombinant proteins, LCABL 13,040–50–60, derived from the PLE3 prophage, identified in the *L. casei* BL23 genome	The LCABL operon in *L. casei* BL23 functions similar to that of the λ-Red system in *E. coli* for genetic manipulation. The process uses plasmids expressing LCABL proteins and the Redγ inhibitor, transforms nisin-induced competent cells with linear DNA cassettes, and integrates target genes through homologous recombination. Antibiotic markers can be removed using Cre-lox, if needed.	High efficiency, precision, and accuracy; the selection marker can be removed via Cre-lox system, if required, which allows for multiple modifications to the bacterial chromosome	Limited effectiveness in other LAB strains; dependence on efficient electroporation; conditions require optimization; antibiotic use required	Gene deletion; gene insertion; point mutations	*Lactobacillus casei* BL23	Deletion of a 167-bp *galK* fragment, insertion of the *gfp* gene, and point mutation of the *rpoB* gene in the *L. casei* BL23 genome	Xin et al.^192^
Cre-loxP system	A site-specific recombinase system. This P1 bacteriophage-based tool comprises two components: two loxP sites that facilitate targeted DNA excision and the Cre recombinase, which catalyzes precise, site-specific recombination events. The Cre recombinase enzyme is utilized to mediate recombination between loxP sites, allowing for controlled genetic modifications.	The Cre-lox system involves use of the Cre recombinase enzyme, which recognizes specific DNA sequences known as loxP sites. These loxP sites are short, 34-bp sequences that consist of two 13-bp palindromic repeats flanking an 8-bp spacer region. When Cre recombinase is expressed in a cell, it binds to loxP sites and catalyzes recombination between them. Depending on the orientation and location of the loxP sites, this recombination can lead to deletion, inversion, or translocation.	Highly effective and precise, enabling marker-less deletions without the need for counter-selection; facilitates sequential deletions and is suitable for large-scale chromosomal modifications; can be used in combination with other genetic tools, such as CRISPR/Cas9	In some cases, a DSB or single-strand nick may fail to activate homology-directed repair, potentially leading to cell death	Gene deletions; gene insertions; gene translocations; gene inversions	*Lactococcus lactis*; *Lactobacillus plantarum*; *Lactobacillus casei*	Deletion of a large-scale genomic region in *L. casei* and *L. lactis* (nonessential regions of the genome)	Lambert, Bongers & Kleerebezem^205^; Xin et al.,^206^; Zhu et al.^207^
Group II introns	Group II introns are self-splicing retrotransposons that serve as site-specific reverse transcription factors with nuclease activity.	The editing process, known as “Retrohoming,” involves the exon binding sequence of an intron recognizing the intronic binding sequence of the target, followed by DNA cleavage and integration through host repair mechanisms.	Functional in bacteria lacking homologous recombination; can be modified to target specific loci; no requirement for introducing antibiotic resistance genes	Limited to gene inactivation through insertion; cannot perform point mutations or scarless knockouts; limited ability to introduce large fragments or modify multiple genes	Gene inactivation; chromosomal-targeted gene disruption; site-specific DNA insertion	*Lactococcus lactis*; *Lactobacillus casei*; *Lactobacillus plantarum*	*L. plantarum thyA* gene inactivation; thymidine-lacking *L. plantarum* used to deliver oxalate decarboxylase into the lumen for treatment of hyperoxaluria and calcium oxalate stone deposition	Wang et al.^208^; Sasikumar et al.^209^

The breakthrough occurred with the discovery of CRISPR-Cas systems in bacteria, which had been originally identified as an adaptive immune mechanism in prokaryotes. Although CRISPR technology was first applied to gram-negative bacteria, its adaptation to gram-positive probiotic bacteria has revolutionized genetic manipulation. The CRISPR-Cas9 system, in particular, offers unprecedented precision in genome editing, allowing the targeting and modification of specific genomic regions with high accuracy. Although applying CRISPR to *Lactobacillus* and *Bifidobacterium* was initially challenging because of their distinct genetic architectures, recent years have seen significant progress in overcoming these obstacles.^197,198,211^ As detailed in Table 3, various CRISPR-based approaches have been developed, including standard CRISPR-Cas9, CRISPR/Cas12a (Cpf1) for multiplex gene editing,^199,200,212^ CRISPRi for gene regulation without DNA alteration,^202,203^ and CRISPR-Cas9D10A nickase systems with reduced off-target effects.^204^ Particularly effective are hybrid approaches combining recombineering with CRISPR-Cas9, which have achieved impressive editing efficiencies of 46–100% (dsDNA recombineering + CRISPR-Cas9) in *Lactobacillus* strains.^194,196^ These tools provide greater control over gene function, enabling the creation of strains with enhanced probiotic properties, such as increased stress tolerance, improved adherence to intestinal cells, and optimized immune-modulating effects.

These developments, along with inducible promoters, more efficient electroporation techniques, and natural competence systems in certain strains, have further facilitated the genetic manipulation of gram-positive probiotics. This has opened the door for exploring new therapeutic applications, including engineering probiotics to deliver therapeutic molecules, break down toxins, and target-specific pathogens in the gut. The key genome editing methods applied to gram-positive probiotic bacteria are presented in Table 3, which provides a comprehensive overview of selected genetic modification tools and techniques used in probiotic bacteria. This summary details the process mechanisms, advantages, disadvantages, and practical applications across various bacterial strains.

Different systems can be combined, especially with CRISPR-Cas systems, where their versatility can be enhanced through integration with complementary technologies. For example, combining the CRISPR-Cas9 system with Cre-lox^213^ increases editing flexibility; pairing the CRISPR-Cas12a system with the RecE/T^214^ system facilitates DNA repair and recombination through RecE/T proteins; linking the Type V-K CRISPR-Cas system with transposons^215^ enables effective genetic modifications, as demonstrated by the ShCAST system; and the CRISPR-dCas9 system can be combined with base editors^216^ to enable precise nucleotide conversions using a modified, non-cutting Cas9 protein.^208^

## Modifications to probiotic strains that increase their protective role in the gastrointestinal tract

Growing knowledge of the pathogenic nature of certain microbes and the critical role that gut microbiota plays in human health has created a challenge in healthcare. Antibiotics remain the primary treatment for bacterial infections; however, their broad-spectrum use often disrupts the gut microbiota, and the overuse of antibiotics in healthcare and agriculture has led to a rise in antibiotic-resistant microbes.^217^ Despite efforts made to counteract these drawbacks, the development of new antimicrobials has been largely unsuccessful, with the challenge of penetrating bacterial cell walls being a key obstacle. As an alternative, enhanced probiotic strains could provide promising supplements or substitutes for traditional antibiotics.^213^ Among the probiotic microorganisms that have been proven beneficial, significant improvements can still be made. Given the mentioned limitations of conventional probiotics, genetic modification tools provide promising avenues for enhancement. As detailed in Table 3, researchers are exploring multiple approaches for improving the capabilities and health-related functionalities of probiotic microorganisms. Several key characteristics of probiotic microorganisms could be optimized, including their ability to adhere to the intestinal lining, survive challenging gastrointestinal conditions, and stimulate the immune system.

Adhesiveness is a key area of development, where the focus lies on engineering strains that can attach more effectively to intestinal walls.^218^ By improving this characteristic, probiotics could establish more stable and lasting interactions within the human digestive system. Survival under challenging GIT conditions is another crucial focus. Strains that can withstand harsh acidic environments resist digestive enzymes, and maintain their structural integrity throughout the digestive process would remain viable and functional when they reach target areas of the intestinal tract.^219^ Research on microbial aggregation has shown that effective probiotic clustering potentially creates more robust defense mechanisms within the gut microbiome.^220^ This extends to immune system stimulation, where enhanced immune response modulation capabilities could lead to improved natural defense mechanisms and inflammatory regulation.^221^ The expansion of production capabilities has led to innovative applications, including the development of probiotics as potential vaccine delivery systems, marking a significant advancement in therapeutic approaches.^222^ Strains that generate health-beneficial enzymes, including bile salt hydrolase and prolyl endopeptidase, contribute to improved metabolic processes and digestion. Bacteriocin production represents a targeted approach to eliminating pathogenic bacteria and creating natural antimicrobial agents within the probiotic framework.^223^ The generation of other metabolites, such as phytoestrogens, can also contribute to host health.^224^ These multiple approaches to enhancing probiotics offer promising strategies for improving gut health and pathogen defense, many of which are discussed in the following sections.

## Improved adherence to the intestinal mucosa

One way in which the adhesion of probiotic strains to mucosal surfaces can be increased is through cloning and expression of adhesins or secretion systems derived from pathogenic species.^225^ Insertion of genes encoding adhesion proteins, such as fimbriae, pili, and mucus-binding proteins, can improve the ability of probiotics to adhere to the intestinal mucosa, thereby enhancing their colonization and interaction with host tissues.^226^ The efficacy of enhancing probiotic binding and adhesion to host cells to improve competitive exclusion strategies has been extensively explored.^218,227–232^ For example, a *Lactococcus lactis* strain engineered to express flagellin derived from a probiotic strain of *Bacillus cereus* CH showed robust adhesion to mucin-coated polystyrene plates *in vitro* .^233^ This strong adherence competitively inhibited the binding of pathogenic *E. coli* and *S. enterica* to the same mucin molecules. The inhibition mechanism involves competitive exclusion, in which probiotic bacteria outcompete pathogens for binding sites on mucin, effectively reducing pathogen colonization and enhancing gut health.^233^

Innocentin et al.^227^ investigated the introduction of *Listeria monocytogenes* internalin A (InlA+) and *Staphylococcus aureus* fibronectin-binding protein A (FnBPA+) or its fibronectin-binding domains C and D (CD+) into *Lactococcus lactis* strains. FnBPA helps bacteria attach to and enter human cells by binding to fibronectin, and InlA enables bacteria to invade cells by interacting with E-cadherin receptors on cell surfaces. Genetic modification of these proteins resulted in increased binding to human epithelial Caco-2 cells and enhanced bacterial internalization. Both engineered strains (*L. lactis* InlA+ and *L. lactis* FnBPA+) exhibited efficient internalization and delivery of DNA within human epithelial cells, demonstrating their potential to enhance probiotic efficacy through improved cellular interaction. The *L. lactis* CD+ strain was internalized less efficiently, but still better than the wild-type strain was.^227^ The InlA and B (InlAB) derived from *L. monocytogenes* were also expressed individually and in combination in *L. casei* ATCC344 using vector-pLP401-T to prevent infection. All modified *L. casei* strains showed increased adhesion to Caco-2 cells compared to that of the unmodified wild-type strain, with the strain expressing both InlA and InlB (Lbc^Inl^ AB) demonstrating the highest adhesion capacity. These recombinant strains significantly reduced *L. monocytogenes* adhesion and invasion in Caco-2 cells, and the cytotoxic effects of Lbc^Inl^ AB were most effective at reducing *L. monocytogenes* adhesion and invasion by 53.6% and 51.7%, respectively, at 24 h.^232^

Additionally, a recombinant *Lactobacillus paracasei* strain was engineered to express *Listeria* adhesion protein (LAP) to combat *L. monocytogenes* infections. LAP is an adhesion factor in *L. monocytogenes* that binds to the host cell receptor, heat shock protein 60, facilitating both listerial adhesion and transepithelial translocation during the intestinal phase of infection. Both the conventional and recombinant forms of *L. paracasei* were separately applied to Caco-2 cell monolayers, which were subsequently stained with Giemsa. The pre-exposure of Caco-2 monolayers to LAP-expressing recombinant *L. paracasei* prior to *L. monocytogenes* exposure resulted in reduced pathogen adhesion and translocation. The wild-type probiotic strain showed no significant decrease in *L. monocytogenes* adhesion to the cell monolayer, whereas the recombinant strain achieved a 60% reduction. When intestinal monolayers were pre-exposed to the recombinant *L. paracasei* probiotic expressing LAP, followed by infection with *L. monocytogenes*, a 44%, 45%, and 46% reduction in adhesion, invasion, and transepithelial translocation, respectively, was observed.^234^ A *Lactobacillus casei* ATCC 334 strain was also engineered to produce LAP from a nonpathogenic strain of *Listeria innocua* and pathogenic strain of *L. monocytogenes* on its surface. This modified *L. casei* strain effectively inhibited the colonization of *L. monocytogenes* by occupying the LAP receptor and prevented systemic transmission of the pathogen. A positive impact can be achieved when maintaining the integrity of the intestinal epithelial barrier through the subsequent relocation of key epithelial junctional proteins (claudin-1, occludin, and E-cadherin), and intestinal immune health enhanced by increasing the number of dendritic, natural killer, and mucosal regulatory T cells in the intestine.^228^

Enhanced *Lactobacillus casei* adhesion was achieved by incorporating an *L. monocytogenes* cell wall-anchoring protein. By introducing the *mub* gene into *L. casei* ATCC 393, the modified strain (*L. casei* pNZ-mub) showed significantly improved adhesion to Caco-2 cells compared with that of the parental strain. Additionally, when combined with a rumen fungal xylanase gene (*xynCDBFV*), the strain (*L. casei* pNZ-mub/xyn) could break down oat spelt xylan and compete better with *L. monocytogenes* for adhesion to Caco-2 cells.^230^

A modified strain of *Lactobacillus acidophilus* engineered with K99 fimbriae derived from enterotoxigenic *E. coli* (ETEC) demonstrated a dose-dependent decrease in ETEC attachment to the porcine intestinal brush border. Chu et al. found that *L. acidophilus* engineered to express recombinant K99 adhesive fimbriae effectively inhibited ETEC adhesion. These results underscore the potential of engineered probiotics to reduce ETEC infections by interfering with bacterial attachment mechanisms in the intestine.^231^ An *L. casei* strain was also genetically modified to produce the ETEC adhesins, K99 and K8872, and its effectiveness as a recombinant probiotic for safeguarding against ETEC infection validated using a mouse model. Oral administration of the modified strain induced substantial mucosal IgA levels in the bronchoalveolar lavage and intestinal fluids, along with a robust systemic IgG response. This engineered probiotic conferred protection to over 80% of vaccinated mice when exposed to a lethal dose of ETEC during subsequent challenge experiments.^235^

The specific adhesins associated with each *L. casei* strain remain unclear. However, efforts have been made to genetically modify this probiotic to express a collagen-binding protein gene that enhances bacterial adhesion.^218^ After the gene encoding collagen-binding protein (*cnb*) was introduced into *Lactobacillus casei* ATCC 393, a significant increase in its ability to adhere to Caco-2 cells was observed when Cnb was placed on the cell surface. Moreover, *L. casei* pNZ-cnb showed higher competitive properties against different pathogens, such as *E. coli* O157:H7 and *Listeria monocytogenes*, for Caco-2 cells than wild type *L. casei* ATCC 393 did. This enhanced adhesion suggests that the modified *Lactobacillus casei* could have improved probiotic properties, potentially leading to better gut colonization and more effective delivery of health benefits.^229^

Previous studies have shown that the presence or absence of pathogenic adhesins on the cell surface can influence the innate immune response of the intestinal epithelium, primarily by affecting the expression and secretion of proinflammatory cytokines and bacterial adhesion and invasion rates.^236,237^ Bearing that in mind, safety concerns often arise when pathogenic surface proteins are introduced into probiotic strains. For instance, the expression of FnBPA in *Lactococcus lactis* enhances its adherence to immobilized fibronectin, but has been associated with valvular lesions in rats. Notably, the adverse effects depend on the route of administration—*L. lactis* caused lesions when injected parenterally, whereas local administration was significantly safer.^227^ Increased adhesion may also promote cellular uptake by intestinal epithelial cells, potentially altering their function and modulating the innate immune response.^234^ Therefore, the safety of expressing pathogenic surface proteins in probiotics must be carefully evaluated, with particular attention to possible inflammatory effects. On the other hand, there are natural processes in which probiotic strains share adhesion-connected genes or gene analogues with pathogenic strains.^238^

## Modulating the host immune system

Probiotic strains possess the ability to manipulate the host immune response. Certain strains can stimulate the production of host AMPs and immunoglobulins, thereby enhancing the ability of the host to fight pathogens. These unique properties of probiotics can be enhanced through genetic modifications. Probiotics can be engineered to enhance the host immune response by increasing the production of anti-inflammatory cytokines, such as IL-10, or by stimulating the production of immunoglobulins, including IgA. For instance, the *Lactococcus lactis* MG1363 strain has been modified to secrete IL-10, which helps reduce intestinal inflammation.^239^ Engineered *L. lactis* effectively reduced low-grade colon inflammation in mice. This strain improved intestinal barrier function by reducing gut hyperpermeability and enhancing the expression of tight junction proteins. It also modulated the immune response by lowering the levels of pro-inflammatory cytokines, leading to a significant reduction in inflammation. Additionally, serotonin levels in the colon, which are often dysregulated under inflammatory conditions, were stabilized, contributing to overall gut homeostasis.^239^

The vaccine candidate was developed using *Lactobacillus casei* CICC 6105 displaying the ETEC 987P fimbrial protein anchored by poly-γ-glutamate synthetase A to trigger mucosal immunity. Administering this recombinant strain to SPF BALB/c mice induced high levels of mucosal IgA in fecal, intestinal, and lung lavage fluids, along with systemic IgG (IgG1, IgG2b, and IgG2a) in serum. The T-cell proliferation assays showed significantly enhanced responses compared with those of controls, with a balanced production of Th1 and Th2 cytokines. The vaccine also provided strong protection (83.3%) against a lethal dose of the virulent ETEC strain, C83916, demonstrating its effectiveness in preventing ETEC 987P infection.^240^

A study investigating low calcium response V (LcrV)-secreting *Lactococcus lactis* demonstrated its significant therapeutic potential for the prevention and treatment of acute colitis in mouse models. The engineered bacteria effectively delivered the immunomodulatory LcrV protein to the colon via oral administration, triggering IL-10 production through a TLR2-dependent pathway. The treatment led to reduced inflammation and tissue damage in the colon, with clear evidence of improved mucosal healing compared with that of the control groups. Notably, the protective effects were found to be IL-10-dependent, as demonstrated by the lack of a therapeutic effect observed in IL-10 knockout mice.^241^

Bacterial flagellins are recognized as antigens that trigger innate immune responses through TLR5 and enhance immune reactions. A recombinant *Lactobacillus casei* strain expressing the flagellar antigen, FliC, derived from *Salmonella* Enteritidis was demonstrated to maintain its ability to stimulate IL-8 production in Caco-2 cells. Furthermore, mice immunized with these recombinant strains produced antigen-specific antibodies and cytokines, wherein this immune response was protective against *S*. Enteritidis infections. Kajikawa and Igimi showed that recombinant *L. casei* displaying flagellin-fusion antigens on its surface induced both innate and acquired immune responses in a mouse model. This included the generation of specific antibodies against the displayed antigen, suggesting the possibility of using engineered probiotics as vaccine delivery vehicles to evoke protective immune responses against bacterial infections, such as by S. Enteritidis.^242^

Similarly, *L. casei* was engineered to express three different lengths of intimin protein fragments (Int261, Int380, and Int527) derived from enteropathogenic *E. coli* (EPEC), which were then used to orally immunize mice. This successfully induced both systemic IgG and mucosal IgA antibody responses. The antibodies produced specifically recognized intimin proteins and demonstrated a notable ability to reduce EPEC adhesion to epithelial cells, with both serum and intestinal antibodies showing inhibitory effects. The *L. casei* strains expressing longer intimin fragments (Int380 and Int527) generated stronger immune responses and produced antibodies with superior inhibitory activity against EPEC adhesion in an epithelial cell culture model.^243^

A novel vaccine delivery system was developed based on the natural ability of *Bacillus subtilis* to form highly resistant endospores. This system includes an urease B (UreB) protein fragment from *Helicobacter acinonychis* that is displayed on the *B. subtilis* spore coat and serves as an antigen against *H. pylori*. The vaccinated mice demonstrated significant protection against *H. pylori* infection, reduced bacterial colonization in the stomach, increased Th1 and Th17 cell populations, effective memory cell generation, and reduced bacterial load.^244^ Another research group engineered *B. subtilis* to express UreB derived from *H. pylori* by fusing it with the spore coat protein, CotC. Oral immunization using a mouse model with these engineered B. *subtilis* spores resulted in a 84% reduction in *H. pylori* stomach bacterial load and the generation of a humoral response.^245^ An *L. lactis* strain was also engineered to express the *H. pylori* adhesin A protein. When administered orally to mice, this engineered strain induced a significant increase in serum IgG levels.^246^ Moreover, an anti-*H. pylori* vaccine was developed by engineering a strain of *Lactococcus lactis* to produce Lpp20, an antigen from *H. pylori*, where the *L. lactis* strain could effectively produce and deliver the Lpp20 antigen, triggering significant immune responses.^247^

Although the most effective treatment currently used against *Clostridium* is fecal microbiota transplantation, *Lactococcus lactis* was engineered to address *C. difficile* infections by expressing the nontoxic fragments of key virulence factors of the pathogen (TcdA and TcdB), demonstrating significant protective effects in animal studies, with up to a 75% survival rate when both fragments were combined. The *L. lactis* oral vaccine promoted strong immune responses, producing both IgG and IgA antibodies capable of neutralizing *C. difficile* toxins *in vitro*. Vaccinated animals showed significantly reduced mortality and less severe symptoms than the control groups did.^248^

## Antibacterial activity

Recombinant LAB have been extensively engineered to combat various pathogens by producing AMPs and targeting specific bacterial signaling pathways. AMPs effective against various pathogens have been successfully produced using *Lactococcus lactis*. The AMPs, A3APO and alyteserin, produced by recombinant *L. lactis* were able to inhibit the growth of pathogenic *E. coli* and *Salmonella* by up to 20-fold without harming the *L. lactis* host. This system, which uses recombinant *L. lactis* to produce and deliver AMPs, could serve as a model for targeting gram-negative pathogenic bacterial populations.^249^

Targeted therapies have been developed to eliminate *H. pylori* while preserving microbial balance in the stomach by bioengineering *L. lactis* to release guided AMPs. Researchers have cloned three AMPs – alyteserin, CRAMP, and laterosporulin – fused to the guiding peptide, MM1, into *L. lactis*. The engineered *L. lactis* selectively killed *H. pylori in vitro*, as qPCR results showed a significant reduction in *H. pylori* abundance compared with that of *E. coli* or *Lactobacillus plantarum*. The chemically synthesized alyteserin and MM1-alyteserin also preferentially inhibited *H. pylori* growth over that of *E. coli*, with MM1-alyteserin having a much higher minimum inhibitory concentration for *E. coli* than alyteserin does, while maintaining the same inhibitory effect against *H. pylori* .^250^

Another example involved combating cholera by engineering *L. lactis* to sense and respond to the presence of *Vibrio cholerae*. As part of its quorum-sensing system, *V. cholerae* produces a signaling molecule called cholera autoinducer 1 (CAI-1), which bacteria use to detect their population density. An *L. lactis* strain was engineered to recognize this molecule, which was achieved by incorporating a gene from *V. cholerae* that encodes the CAI-1 receptor (CqsS). In the presence of CAI-1, this receptor activates a signaling pathway in the engineered *L. lactis* strain. Once *L. lactis* detects *V. cholerae* (via CAI-1), it triggers a response that suppresses the pathogen. Mice treated with engineered *L. lactis* and then exposed to *V. cholerae* showed a significant reduction in cholera symptoms compared with that of untreated mice.^251^

Genes crucial for autoinducing peptide I (AIP-I) detection were introduced into *Lactobacillus reuteri* by incorporating the AgrC-AgrA two-component system from *S. aureus*. When AIP-I binds to AgrC, it activates AgrA, which then triggers the expression of a reporter gene in *L. reuteri*. This reporter gene activation produces a detectable signal, indicating the presence of *S. aureus*. To measure this, AgrA activation was linked to the production of a fluorescent reporter protein. Nanomolar to micromolar levels of bacterial signaling molecules can be detected by *L. reuteri*. Upon sensing AIP-I, the modified *L. reuteri* emits a fluorescent signal, effectively serving as a biosensor for the early and precise detections of *S. aureus* infections.^252^

To combat multidrug-resistant *Enterococcus* species, engineered *Lactococcus lactis* systems with two distinct activation mechanisms have been used. In one approach, *L. lactis* was engineered to detect the *E. faecalis* pheromone, cCF10, which triggers the release of specific bacteriocins that effectively kill multidrug-resistant *E. faecalis*, achieving a 10,000-fold reduction in bacterial counts.^253^ The second strategy targeted *E. faecium*, which does not produce the cCF10 pheromone, by engineering *L. lactis* to produce bacteriocins under the control of a chloride-inducible promoter that is naturally activated in the gut environment, demonstrating the potential of these systems as alternatives to traditional antibiotics for treating resistant enterococcal infections.^254^

*Lactococcus lactis* was engineered with albumin-binding domains (ABD variants S1B22 and S1B26) specifically designed against the Shiga toxin 1 B subunit (Stx1B). This resulted in successful surface expression through fusion with the Usp45 secretion signal and the AcmA peptidoglycan-binding domain. The engineered bacteria effectively bind to Stx1B via the ABD variants, disrupting the retrograde transport of Stx1B to the Golgi apparatus.^255^

The genetically engineered *Lactobacillus casei* strain, LC + myosin cross-reactive antigen (mcra), which overexpresses the *mcra* gene, demonstrated significant antimicrobial activity against *Campylobacter jejuni*. LC + mcra completely inhibited *C. jejuni* growth within 48 h and reduced its adherence to and invasion of HD-11 and HeLa cells while also significantly altering *C. jejuni* virulence-related gene expression. Similarly, LC + mcra cell-free culture supernatants and their combination with peanut flour effectively reduced *C. jejuni* growth and pathogenicity.^256^ These findings, along with the enhanced production of conjugated linoleic acid, highlight LC + mcra as a promising approach for controlling enteric pathogens, including *Salmonella* Typhimurium and enterohemorrhagic *E. coli*, while promoting anti-inflammatory and anti-infective effects.^257^

## Escherichia coli Nissle 1917: a well-characterized probiotic and promising platform for therapeutic engineering

Our work primarily focused on gram-positive bacteria; however, it is worth mentioning that the probiotic EcN strain is a well-studied probiotic with numerous applications in the treatment of intestinal diseases, particularly inflammatory bowel disease and ulcerative colitis.^34^ For nearly a century, EcN has been used as a medicinal product with demonstrated efficacy and safety.^258^ Genetic modification of gram-negative bacteria is generally better established, which makes EcN an ideal candidate for advanced engineering and a robust platform for developing enhanced therapeutic applications. The versatility of engineered EcN is evident from its diverse applications in the fight against pathogens. When engineered to detect *Pseudomonas aeruginosa* signaling molecules, *N*-acyl homoserine lactone (AHL), the modified probiotic released targeted antibiotics and antibiofilm enzymes in response to AHL. This approach has been tested on two models: *Caenorhabditis elegans* and mice, where it showed both preventive and therapeutic effects. The engineered strain significantly reduced *P. aeruginosa* colonization in the gut, effectively suppressing infection and demonstrating its potential as a targeted treatment for gut infections.^259^ EcN was also designed to detect other molecules, such as tetrathionate, a molecular signal produced during gut inflammation, and respond by producing the AMP, microcin H47. EcN demonstrated selective antimicrobial activity, inhibiting *Salmonella* growth only when tetrathionate was present at a concentration of 1 mM, but showed no inhibition in its absence. The engineered strain exhibited a significant competitive advantage over *Salmonella* in direct competitive assays in the presence of tetrathionate and effectively responded to inflammatory signals and suppressed pathogen growth *in vitro*.^260^

By producing AMPs, such as enterocin A, enterocin B, and hiracin JM79, EcN was able to target and eliminate the two most common vancomycin-resistant *Enterococcus* (VRE) species, *E. faecalis* and *E. faecium*. Administration of engineered EcN significantly reduced the abundance of these *Enterococcus* species in the feces of male BALB/cJ mice. These modifications enhanced the protective role of EcN in the GIT. Enterocin A, derived from *Enterococcus* species, directly targets VRE, whereas bacteriocin 43 inhibits other enterococci, including antibiotic-resistant strains. This approach not only effectively targets VRE but also highlights the potential for adapting these engineering strategies against other drug-resistant pathogens in the GIT, such as *Clostridium difficile* and multidrug-resistant *Klebsiella pneumoniae*.^261^ The expression of CAI-1 by EcN significantly increased mouse survival rates and reduced cholera toxins binding in the intestines when administered before *V. cholerae* infection. Earlier work by Duan and March^262^ showed that this engineered *E. coli* strain could interrupt the expression of *V. cholerae* virulence genes *in vitro*.^263^ When engineered to produce 3-hydroxybutyrate (3HB) for prolonged synthesis in the colons of colitis-afflicted mice, EcN enhanced the gut environment and contributed to disease treatment. The presence of the modified strain resulted in a significant increase in 3HB and SCFA levels by 8.7-fold and 3.1-fold, respectively, compared with those of the wild-type strain. The sustained presence of 3HB in the gut encouraged the growth of beneficial microbes, particularly *Akkermansia* spp. (a mucin-degrading probiotic), which expanded from 2% to over 31% of the microbiome.^264^

## Other potential applications

This review focuses on engineered probiotics that support the host in combating enteric pathogens, but it is important to note probiotic strains’ potential in other therapeutic areas. Engineered probiotics are being developed to address a wide range of conditions, including metabolic disorders, cancer, and inflammatory diseases.^265,266^ Unlike conventional engineered strains, they can be precisely tuned to respond to specific disease biomarkers – sensing inflammation-associated molecules like nitric oxide or tetrathionate in inflammatory bowel disease (IBD) or glucose levels in diabetes – and respond with proportional therapeutic outputs. Engineered probiotics are emerging as a promising therapeutic approach for IBD. These genetically modified bacteria can produce anti-inflammatory cytokines, antimicrobial peptides, and other bioactive compounds to treat intestinal inflammation.^265,266^ Studies have shown that engineered probiotics can reduce disease activity and ameliorate intestinal damage in IBD animal models.^267^ Compared to conventional probiotics, engineered strains offer more precise disease management and potentially greater efficacy.^268^ Clinical trial involving 10 patients with Crohn’s disease demonstrated that *L. lactis* engineered to produce IL-10 was safe and associated with a reduction in disease.^269^ Multi-strain probiotics containing specific bacterial species may improve glycemic control in type 2 diabetes patients.^270^ GM *Lactobacillus plantarum* expressing GLP-1 (glucagon-like peptide-1) was demonstrated to be effective in alleviating diabetic symptoms and regulating intestinal microbiota in mouse models.^271^ Similarly, oral administration of engineered *Lactobacillus gasseri* producing GLP-1 can reprogram intestinal cells into insulin-secreting cells, significantly improving glucose tolerance in diabetic rats.^272^ Administration of engineered *E. coli* Nissle 1917 expressing N-acyl-phosphatidylethanolamines resulted in significant improvements in markers of cardiometabolic disease in mice, including reduced food intake and weight gain, lower plasma cholesterol, and improved glucose tolerance.^273^ Another significant clinical advancement of engineered EcN demonstrated the development of a synthetic live bacterial therapeutic, SYNB1618, designed to treat phenylketonuria (PKU) by degrading phenylalanine in the gastrointestinal tract.^274^ Engineered EcN strains have shown promising safety profiles and functionality in Phase 1/2a clinical trials, metabolizing phenylalanine in the GI tract with the potential to reduce blood phenylalanine levels in PKU patients.^275^ Beyond these examples, engineered probiotics show promise for treating colorectal cancer (CRC). Engineered probiotics have shown promise in reducing tumor burden by releasing P8 therapeutic protein using *Pediococcus pentosaceus* as a drug delivery system. Studies have demonstrated that these probiotics can modulate gut microbiota, potentially alleviating dysbiosis associated with CRC.^276^ As highlighted in comprehensive reviews,^19,277^ these expanded applications represent the remarkable versatility of synthetic probiotics as next-generation therapeutics. However, despite numerous studies demonstrating efficacy of animal models, clinical translation remains a significant challenge.^19^

## Future prospective

Although the use of probiotics is common, and studies have shown promising results, the application of genetically engineered bacteria in human therapy remains experimental, requiring extensive clinical trials to establish their safety and efficacy. Probiotic bioengineering is a field with remarkable potential; however, it still faces considerable technical and regulatory challenges that must be overcome.

The next generation of engineered probiotics promises to extend far beyond current capabilities, offering therapeutic solutions through intelligent sensing and response systems. These advanced probiotics could simultaneously detect multiple disease biomarkers and coordinate therapeutic responses through bacterial communication networks, essentially functioning as “smart” living therapeutics. In addition, they could change dynamically with changes that occur in the gut environment when detecting signals produced by the host or pathogen-related molecules. Probiotics could function as personalized microbiome medicines, and real-time monitoring and dynamic adjustment of probiotic therapies could be tailored to individual genetic and microbiome profiles. Integration with nanotechnology could enhance delivery precision, whereas advanced encapsulation systems could ensure targeted therapeutic delivery to specific gut regions. Novel applications may extend beyond traditional gut health to other areas that include mental health interventions through engineered neurotransmitter production, the prevention of age-related cognitive decline, and environmental health applications.

These advances will require the development of more efficient genetic modification techniques, modeling systems, and an understanding of the complex interactions that occur among the host, pathogen, and microbiome. This includes metabolic crosstalk between engineered strains and native microbiota. Future research should address how engineered strains can enhance colonization resistance against pathogens while preserving beneficial members of the microbiota. Moreover, these advances must be balanced against safety considerations, including the prevention of HGT, stability of genetic modifications, and potential impacts on microbiome homeostasis. Meeting the regulatory requirements for human consumption requires the development of food-grade selection markers and containment strategies, along with comprehensive safety assessment protocols that evaluate both the short- and long-term effects on host health and microbiome composition.

## Data Availability

No data were used for the research described in the article.
